# Full-length genome sequence of Ntaya virus

**DOI:** 10.1007/s11262-012-0825-7

**Published:** 2012-09-21

**Authors:** Meik Dilcher, Amadou A. Sall, Frank T. Hufert, Manfred Weidmann

**Affiliations:** 1 Department of Virology, University Medical Center Goettingen, Kreuzbergring 57, 37075 Goettingen, Germany; 2 Institut Pasteur de Dakar, 36 Avenue Pasteur, B.P. 220, Dakar, Senegal

**Keywords:** Ntaya virus, Flavivirus, 454 Pyrosequencing

## Abstract

Presentation of pyrosequencing data and phylogenetic analysis for the full genome of Ntaya virus, type virus of the Ntaya virus group of the *Flaviviridae* isolated in Cameroon in 1966.

The Flavivirus Ntaya virus (NTAV) was originally isolated from mosquitoes in Uganda in 1951 [1] and serologically determined as the type virus of the Ntaya virus group within the Flaviviridae [2]. Serosurveys have detected NTAV activity in migratory birds and domestic animals in Romania [3–6]. Antibodies against NTAV in travellers from Africa indicate transmission in Uganda, Cameroon, Democratic Republic of Congo, Kenya, Nigeria and Zambia. Clinical evidence indicated neurological manifestations of disease [7].

A lyophilised 10 % suckling mouse brain suspension of NTAV isolate IPD/A of the CRORA collection at the Institute Pasteur Dakar collected in Cameroon in 1966 was passaged twice on Vero E6 cells in 175-cm^2^ tissue culture flasks (DMEM, 2 % FBS, 2 mM glutamine, 10 mM penicillin, 10 mM streptomycin and 20 mM HEPES) at 37 °C and 5 % CO_2_. At 90–100 % CPE (12 dpi), culture supernatants of infected cells were collected, and purification and RNA extraction were performed as described [8]. In order to determine the termini, a self-complimentary 3′-FLAC adapter was ligated to the 3′ end and a 5′-RACE adapter was ligated to the 5′ end of the +ssRNA genome prior to pyrosequencing as described [9]. The complete genome was determined in a pool of seven MID-tagged virus libraries in one pyrosequencing run. Bioinformatic analysis was performed as described [8]. The genome size was 10,891 bp (GenBank JX236040). 93 % of 4,730 reads were specific for NTAV (coverage 116-fold). The NTAV genome was assembled by reference mapping and showed the highest nucleic acid identity (77 %) to strains of Bagaza virus (10,284–10,941 bp).

The NTAV polyprotein (3,427 aa) shows the typical modular flavivirus structure of capsid protein C, polyprotein propeptide, precursor glycoprotein prM, glycoprotein E, non-structural proteins NS1, NS2A, NS2B, peptidase S7 and NS3 serine protease, P-loop-NTPase, non-structural proteins NS4A, NS4B, AdoMet-MTase and RNA-directed RNA-polymerase NS5 (Fig. 1b). Phylogenetic analysis confirms placement of NTAV in a distinct group to which it was assigned as type species by serological methods. High bootstrap values support this placement (Fig. 1a). Molecular methods may now be developed to investigate the role of NTAV role in acute disease in humans and/or animals.

**Fig. 1:**
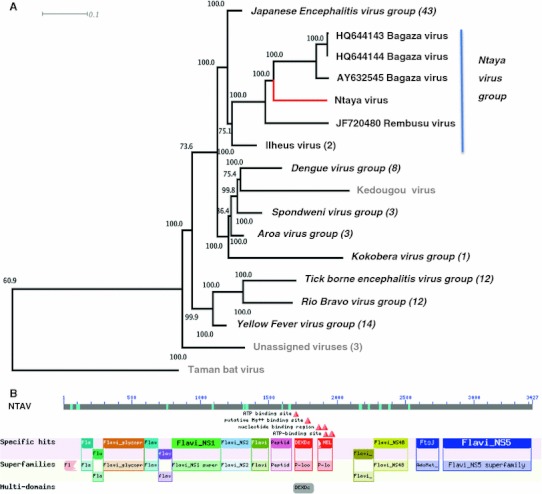
**a** Neighbour-joining phylogenetic analysis of 88 full-length Flavivirus genomes was performed using ClustalW and a 1,000-fold bootstrap approach. Bootstrap values are given in percentage. *Flavivirus group,* Flavivirus, unassigned Flavivirus in *grey* (number of sequences included in the analysis). Ntaya virus on *red* branch. **b** Genome structure of Ntaya virus determined with the Conserved Domains search tool of NCBI [10], http://www.ncbi.nlm.nih.gov/Structure/cdd/cdd.shtml (Color figure online)
